# The Practice of Surgery

**Published:** 1914-09

**Authors:** 


**The Practice of Surgery.** By RUSSELL HOWARD, M.S., F.R.C.S. Pp. viii, 1227. London : Edward Arnold. 1914. Price, 21s. net.

This is a good book, and supplies a real need. It is very clear and concise, omits freaks and varieties, and is not overburdened with pathology or details of operations which no one but an expert should attempt. It aims at providing a textbook for the student reading for the colleges or a pass degree, and we think he will find it simpler and clearer than some of its competitors. It is eminently practical, and devotes adequate attention to everyday surgery. It is well up to date. The illustrations are numerous, and with a few exceptions (figures 30, 341, 443) clear and artistic.

The points upon which we venture to differ from the author, or where we think the book could be improved, are all comparatively trifling. We are not sure that a Von Pirquet's test is " of great value if the reaction is positive," at any rate in adults ; " heterodox mitosis " (!) is presumably " heterotype " ; adrenalin and pituitary extract are not " rapidly oxidized " after injection, as far as we know. We think it should be mentioned that death has followed injection with salvarsan. The author still supports the now somewhat discredited theory that cutting the thoracic duct is a grave injury, and he gives Albrecht's explanation of cleft palate. The gastric HCl. is erroneously given as 2 per cent. (p. 662). We think the interesting condition called traumatic asphyxia not too rare to merit a word or two.

